# A Single-Step Route to Single-Crystal Molybdenum Disulphide (MoS_2_) Monolayer domains

**DOI:** 10.1038/s41598-019-40893-z

**Published:** 2019-03-11

**Authors:** Hamid Khan, Henry Medina, Lee Kheng Tan, Wengweei Tjiu, Stuart A. Boden, Jinghua Teng, Iris Nandhakumar

**Affiliations:** 1 School of Chemistry, University of Southampton, Southampton, SO17 1BJ UK; 2 Institute of Materials Research & Engineering (IMRE), A*STAR, 2 Fusionopolis Way, Singapore, 138634 Singapore; 3 Electronics and Computer Science, University of Southampton, Southampton, SO17 1BJ UK

## Abstract

We report a simple, single-cycle synthetic method for forming highly-crystalline, micron-sized monolayer domains of phase-pure MoS_2_. This method combines liquid chemistry with discrete, layer-by-layer deposition from a novel Mo precursor. Single-crystalline MoS_2_ with domain sizes up to 100 μm have been obtained and characterised by optical and electron microscopy as well as Raman and photoluminescence spectroscopy.

## Introduction

There has been a tremendous amount of interest in 2D layered transition metal dichalcogenides (TMDCs) such as molybdenum disulphide (MoS_2_) for a wide range of electronic and optical applications^1–7^. However, to fully realise the potential of MoS_2_ and other TMDCs for these applications, more efficient and scalable synthetic routes need to be developed that achieve single-crystal growth. Approaches such as mechanical and liquid exfoliation have so far demonstrated only limited scalability^8–10^, while physical and chemical vapour deposition (P/CVD) yield good-quality micron-scale single crystals but larger films exhibit defects arising from polycrystallinity^4,6,11–25^. Atomic layer deposition (ALD)^26–28^, on the other hand, permits precise thickness control because precursors are deposited discretely and the technique avoids the environmental and safety issues inherent to organometallic precursors^19^. ALD of MoS_2_ was first demonstrated by Tan *et al*.^29^. Liquid-based techniques are used to react precursors at solution interfaces, which benefits from low cost and large volumes^30–34^. However, they are generally difficult to control and better suited to nanoparticles or porous materials^35^.

Herein we report a single-substrate^36^, single-cycle route to high-quality, single-crystal monolayer domains of MoS_2_ with sizes up to 100 μm as evidenced by optical microscopy (OM), Raman spectroscopy, Photoluminescense (PL), scanning electron microscopy (SEM) and scanning transmission electron microscopy (STEM). The novelty of our method lies in the use of ammonium heptamolybdate tetra hydrate ((NH_4_)_6_Mo_7_O_24_.4H_2_O) as a Mo precursor which is deposited onto substrates in a single dip-coating step from a heated solution. This is followed by sulphurisation inside a tube furnace. Our approach is low cost and benefits from a readily-available precursor and to the best of our knowledge has never been reported previously. Table 1 summarises the ways in which our method differs from, and improves on, other reported methods from the literature.

**Table 1:** Improvements on the literature by our method.

Prior art	References	Improvements with our method
Surface modification or seeding	refs.^13,18,19^	Bare, untreated SiO_2_ surface gives good-quality crystals
Two-substrate method	ref.^33^	Single substrate improves efficiency
Complex precursors	ref.^19^	Commercially available precursors reduce cost and complexity
Multiple deposition cycles	refs.^29^	Single cycle improves efficiency
Polycrystalline film	refs.^4,6^	Large single crystals better for optoelectronic applications
Long/multistep annealing	refs.^13–15,18,19,22^	Single, short anneal gives good-quality crystals

## Methods

The substrates used throughout were thermally-grown 285 nm SiO_2_ on Si-(100) and were purchased from University Wafer. The wafers had a thickness of 500 μm and a resistivity of 0–100 Ωcm. Before each experiment, wafer dice were cleaned by sequential sonication (15 min.) in acetone, water and isopropyl alcohol (IPA), and dried in nitrogen. In some cases, the final wash with IPA was followed by a 30-minute water dip to improve hydrophilicity and aid the precursor adsorption process from aqueous solution.

Thermal decomposition of (NH_4_)_6_Mo_7_O_24_.4H_2_O (CAS number 12054-85-2, purchased from Sigma-Aldrich at ≥99.999% purity), was studied by thermogravimetric analysis (TGA, Universal V4.5A TA Instruments). (NH_4_)_6_Mo_7_O_24_.4H_2_O solution was manually dip-coated onto cleaned wafer dice at a range of temperatures between 40 and 90 °C. Reaction conditions were achieved by suspending the dice in a pre-heated solution and maintaining the temperature throughout the deposition time. Dice were pulled out after various deposition times.

Precursor-coated substrates were sulphurised with sulphur flakes (CAS number 7704-34-9, purchased from Sigma-Aldrich, purity ≧ 99.99%). For this the substrates were placed inside a quartz tube within a tube furnace. The quartz tube was placed in the middle of the reaction tube, nearest to the thermocouple. The sulphur flakes were placed in a ceramic boat upstream of the substrate container in the cooler part of the reaction tube. Different annealing temperatures were tested, and some samples were post-annealed.

Regions of interest were identified by optical microscopy using an Olympus DX51 microscope with top-view imaging (DP12 digital camera system). Field-emission SEM (FE-SEM, JEOL FESEM6700x) was used to characterise morphology. Morphological and lateral domain size data were obtained by SEM and were complemented by thickness measurements from AFM (JPK NanoWizard® NanoOptics) in force modulation mode. Raman microscopy (Renishaw RL532C10 InVia microscope) was used to obtain domain thickness information with a 532 nm 500 mW excitation source and a grating of 1800 lines/mm. Room-temperature PL spectra and maps were collected (WITEC photon scanning tunnelling microscope) with a 532 nm excitation wavelength for a quantitative measure of crystal quality. An STEM (FEI Titan 80–300) at an operating voltage of 200 kV was used to visualise the nanostructure of the MoS_2_ monolayers. For SEM, samples were characterised on a 35 mm standard sample holder with carbon tape. The material was not sputter-coated with a conductive layer. Images were acquired in secondary electron imaging mode, at an acceleration voltage of 5.0 kV and working distances of 6.4–8.3 mm. In this mode, charging was low-level and acceptable.

TEM samples were prepared by the following method: The as-synthesised films were cleaned by dipping in 2 M aqueous KOH for 5 min. at room temperature. A thin layer of PMMA-950 was spin-coated onto the samples at 2000 rpm for 30 s. The PMMA-coated films were then etched in 2 M aqueous KOH at 75 °C. The material films detached from the SiO_2_/Si substrate after 20 min. The detached PMMA-coated films were collected on TEM grids, and the organic coating was removed by an acetone dip.

## Results and Discussion

**Figure 1:**
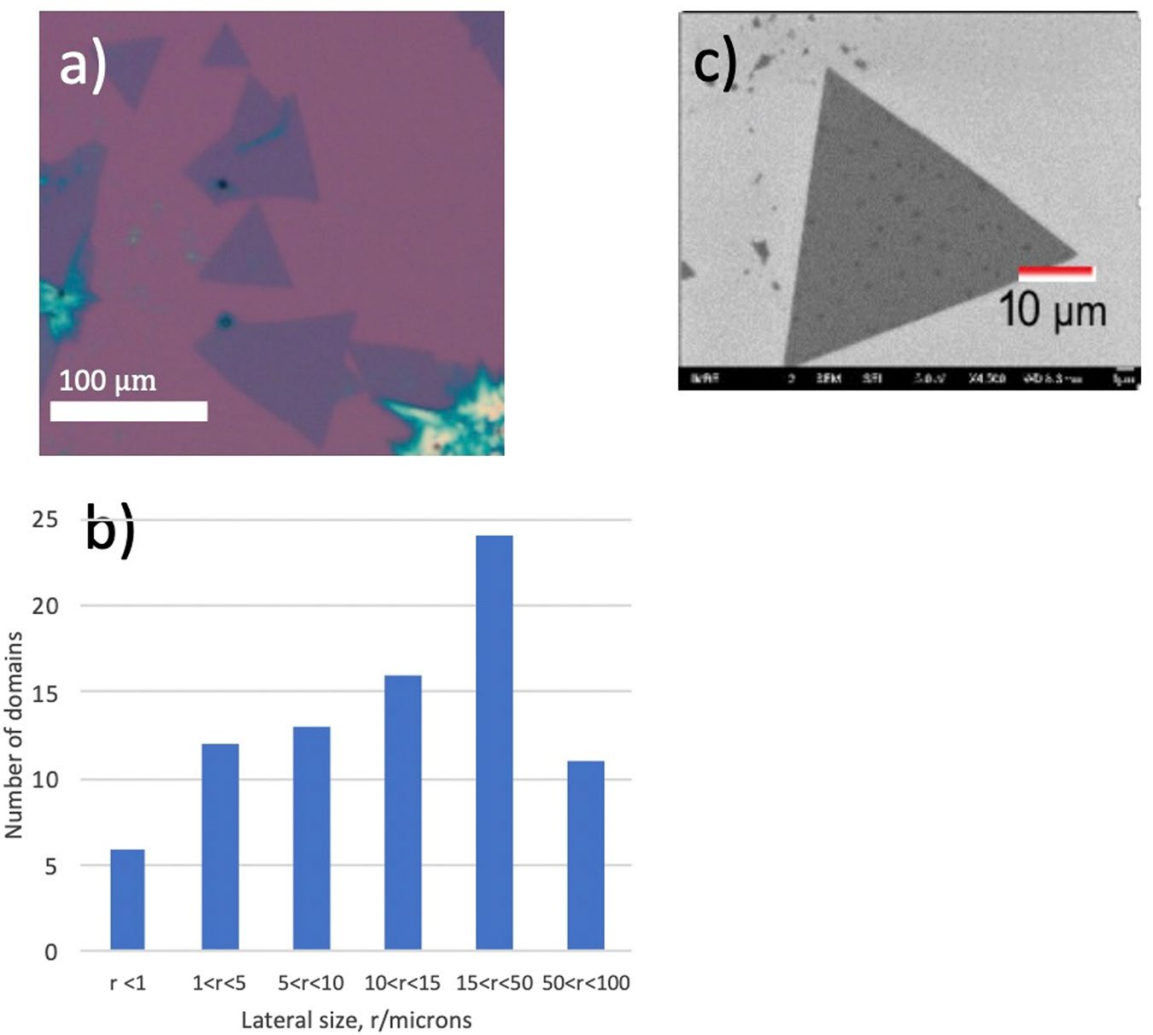
(**a**) Optical image showing regions of coverage of MoS_2_ thin films obtained from just 15 minutes of dip-coating at 80 °C and ten minutes of annealing in sulphur; (**b**) lateral size distribution of as-synthesised domains without post-annealing. (**c**) FE-SEM image showing triangular morphology of ML, micron-sized domain.

Optical images as shown in Fig. 1(a) demonstrate MoS_2_ growth on bare dice from 0.13 g mL^−1^ aqueous solutions of (NH_4_)_6_Mo_7_O_24_.4H_2_O in as little as 15 minutes which is evidenced by the triangular-faceted morphology well-known in CVD-type syntheses of 2D sulphides. Increasing the concentration up to 0.2 g mL^−1^ yielded mostly bulk and few-layer films, so dilute precursor solutions are preferable. Water was the best solvent because the precursor had the best solubility therein. Acetone and ethanol-water mixtures were found to be unsuitable solvents for ammonium molybdate as the surface coverage was very poor. Table S1 in the supplementary information provides a summary of dip-coating parameters.

FE-SEM as shown in Fig. 1(c) reveal straight-edged triangular morphologies that represent monolayers as well as multi-layer terraces^4,5,7,37,38^. These morphologies are consistent with previous reports of single crystal growth. A straight-edged morphology indicates that Mo-terminated single domains were synthesised by our method (S-rich conditions result in Mo-limited kinetics), whilst S-terminated crystals would result in curved edges^4,7^. A layer thicknesses of 0.6–2.5 nm could be determined by AFM line profiling, consistent with 1–4L growth^5,12,14^.

A selection of 50 domains was sampled from six regions of growth to give a representative lateral domain size distribution (see Fig. 1(b)) estimated from OM. The size distribution was obtained after sulphurisation of two good samples that were dip-coated at 80 °C, and confirms that large domains were obtained by our method, with a modal range of 15–50 μm and several single crystals exceeding 50 μm. The largest crystals had diameters of ~100 μm (see Fig. 1(a)) and provided evidence of large-scale growth. At deposition temperatures below 50 °C and above 80 °C, the precursor did not sufficiently cover the substrate. This resulted in poor sulphurisation and a lack of good-quality domains. It is unclear why high-temperature dip-coating was ineffective. It is possible that the kinetic energy of precursor molecules was greater than the adsorption energy at temperatures near the water boiling point leading to desorption. There might have also been a complementary effect from re-dissolution of the precursor at elevated temperatures.

A clear relationship exists between dip-coating temperature and lateral grain size: The largest crystals (r_max_ ~ 100 μm) were grown by dip-coating at 80 °C. At 70 °C, crystals were much smaller (r_max_ ~ 20 μm) whereas at 50–60 °C the majority of crystals was found to be sub-10 μm in diameter. This trend may be attributed to poorer precursor adsorption at lower temperatures. Our results suggest that dip-coating temperature was the key parameter in lateral domain size control, with a smaller contribution from dip-coating time, and that layer thickness was dependent on precursor solution concentration.

Sulphurisation parameters can be found in Supplementary Table S2. A previous report indicated a trade-off between the lateral and vertical diffusion rates of sulphur vapour over and into the precursor^15^. At low temperatures, the rate of mass diffusion of sulphur vapour over the sample exceeds the rate of mass diffusion into the precursor, which results in poor coverage. This situation is reversed at high temperatures where coverage is improved but films are thicker. However, other authors have found no correlation between synthesis temperature and sample quality^20^. In our work, optimal sulphurisation parameters were identified as comprising ten minutes of annealing at 800 °C under nitrogen (100 sccm) with 1600 mg sulphur flakes (see Supplementary Fig. 1 for TGA of the precursor, which indicates why 800 °C is a good sulphurisation temperature and Supplementary Scheme S3). In contrast, sulphurisation at 600 °C yielded poorer coverage and morphology.

**Figure 2:**
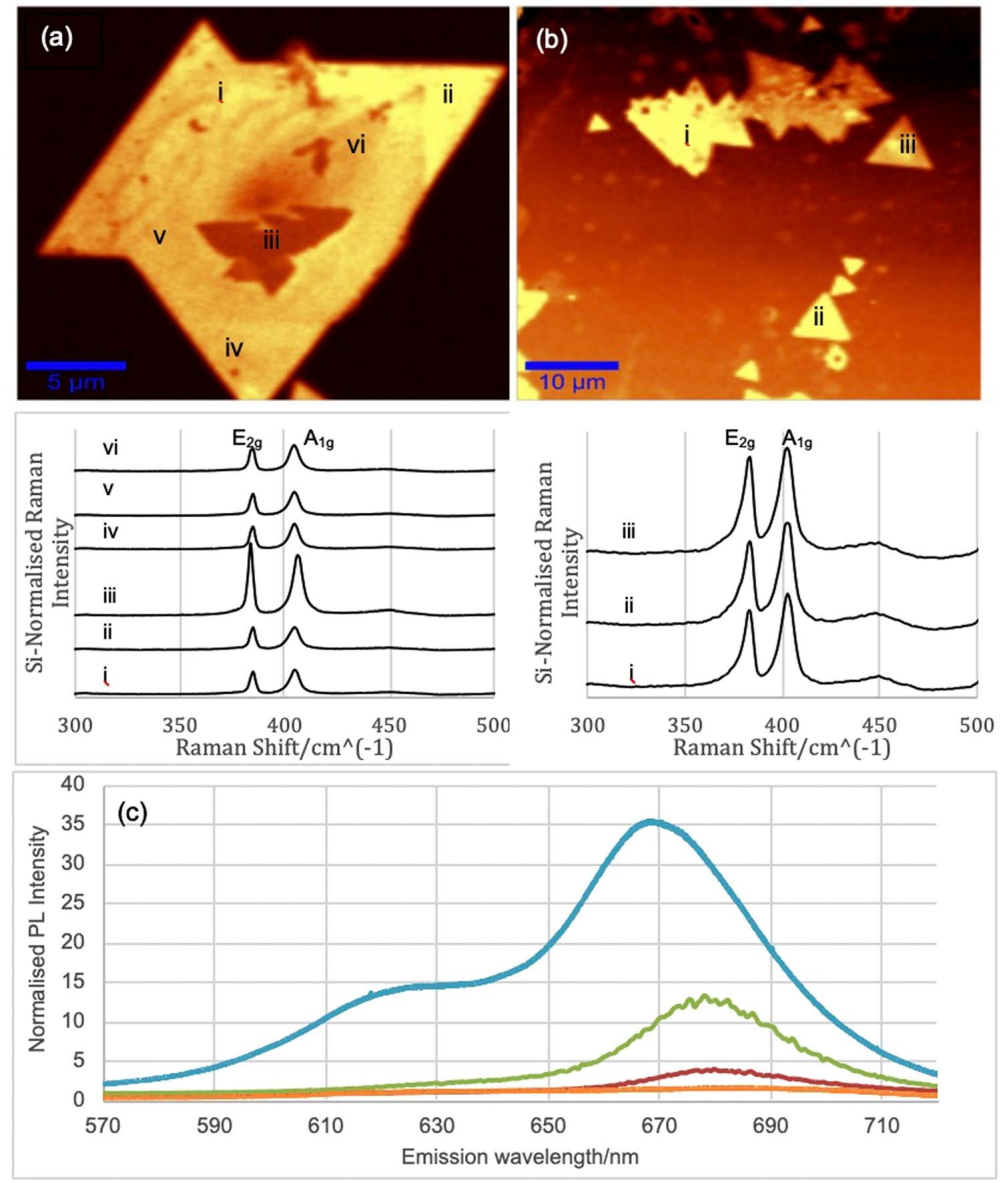
PL maps of selected areas on domains confirm crystal quality. Corresponding Raman spectra for each PL-active domain are shown below. These were acquired using the 50x objective. The maximum laser power at source was 500 mW. The absence of a Raman peak at 335 cm^−1^ confirms phase purity. (**a**) 20 × 20 μm field of view (FOV). (**b**) 50 × 50 μm FOV. (**c**) PL spectra from regions of different thickness acquired at 532 nm excitation; spectra are normalised to the laser line; (blue) intense peak at 668 nm corresponds to A-exciton emission at 1.86 eV in the ML limit; (green) redshift of the peak to 677 nm observed in the 2L limit along with large decrease in intensity; (red) further redshift in 3L limit yields weak emission at 680 nm; (orange) negligible PL in the bulk (>5L) limit.

There are two phases of monolayer MoS_2_: the semiconducting 2H phase, which has a direct bandgap, and the metallic 1T phase. These two phases can be distinguished by Raman spectroscopy and PL. The 2H phase possesses a hexagonal lattice and exhibits two characteristic Raman modes of MoS_2_ which are the in-plane E_2g_ and the out-of-plane A_1g_, at ~38 cm^−1^ and ~40 cm^−1^, respectively. The 1T phase possesses a tetragonal lattice and exhibits the same Raman modes, along with an additional intense mode at ~33 cm^−1^ that is forbidden in the 2H phase. Given that the 2H is the thermodynamically stable phase, whilst the 1T is metastable, it is expected that any 1T-MoS_2_ synthesised in the sulphurisation process was converted to 2H-MoS_2_ during annealing. This is confirmed by both the absence of a peak at 335 cm^−1^ in the Raman spectra and the presence of strong PL as in Fig. 2 (the 1T phase, being metallic, does not generate PL).

The observed Raman shifts exhibit a well-known layer dependence. Specifically, CVD-synthesised MoS_2_ monolayers on SiO_2_/Si typically exhibit characteristic vibrational modes with ${\rm{\Delta }}\bar{{\rm{\upsilon }}}$ around 19.0–20.5 cm^−1^ ^6,12–14,20^. We have observed this range of ${\rm{\Delta }}\bar{{\rm{\upsilon }}}$ (Table 1).

The Raman spectrum of point iii in Fig. 2(a) suggests a layer thickness of 2–3L. The thickness is corroborated by a drop in PL intensity at that point. Variations between ${\rm{\Delta }}\bar{{\rm{\upsilon }}}$ of discrete monolayer domains is attributed to strain-related effects^39,40^. Thermal diffusion also has a role in the shifting of vibrational energy^41^, and for this reason laser power was controlled at 10% of maximum output. Generally, characteristic peak separation increases with layer thickness up to the 5L limit; bulk MoS_2_ exhibits ${\rm{\Delta }}\bar{{\rm{\upsilon }}}$ ≥ 25.0 cm^−1^.

Important information about material quality can be obtained from the ratio of peak intensities, I_A_/I_E_, between the A_1g_ and E_2g_ modes^42^. Cases in which (I_A_/I_E_) < 1, as shown in Table 2, indicate doping of the material, and this likely comes from oxide impurities relating to the substrate. This can explain the reduction in PL intensity across different monolayer regions of domains in Fig. 2(a,b). Another possible explanation is the existence of grain boundaries, which are known to quench PL by up to 50%. With the exception of point 2(a) iii, the domains sampled show good uniformity.

**Table 2:** Raman modes of PL-active domains in Fig. 2 and data extracted thereof.

Region	E_2g_/cm^−1^	A_1g_/cm^−1^	${{\boldsymbol{\Delta }}\bar{{\boldsymbol{\upsilon }}}{\boldsymbol{/}}{\bf{\text{cm}}}}^{{\boldsymbol{-}}{\bf{1}}}$	Layer thickness	$\frac{{{\bf{I}}}_{{\bf{A}}}}{{{\bf{I}}}_{{\bf{E}}}}$
Key Raman data of domain in Fig. 2(a)					
i	384.9	404.3	19.4	1L	1.08
ii	384.9	404.3	19.4	1L	1.17
iii	383.6	405.6	22.0	2–3L	0.83
iv	384.9	404.3	19.4	1L	1.13
v	384.9	404.3	19.4	1L	1.08
vi	384.9	404.3	19.4	1L	1.01
Key Raman data of domains in Fig. 2(b)					
i	385.0	404.2	19.2	1L	1.08
ii	385.0	404.1	19.1	1L	1.10
iii	385.0	404.1	19.1	1L	1.05

PL maps as shown in Fig. 2(a,b) reveal domains that are mono/few-layer and have adopted the 2H phase, while 2H produces strong PL. This is corroborated by the Raman peaks (cf. Table 1), which are consistent with production of the 2H phase^43^.

Figure 2(c) shows representative PL spectra from MoS_2_ regions of different thicknesses, including points ii and iii. The A-exciton emission in the ML case (blue) represents a nine-fold improvement in intensity over the corresponding 3L peak (red), and a threefold improvement over the 2L (green) peak. This compares well to previous reports on PL of mechanically-exfoliated samples. By conducting Raman and PL experiments on different areas of the sample, we could not detect significant variation in the Raman and PL signatures. In addition, we have conducted Raman mapping on different samples as detailed in Supplementary Fig. S2, however PL mapping was preferred over Raman mapping due to the higher sensitivity of PL as a function of number of layers, giving a clear image of the overgrown areas.

Our data also compares well with the CVD results from the literature. For instance, Jeon *et al*. obtained a roughly fourfold improvement in ML-PL intensity over the 2L condition^1^. Our spectra show the expected redshift in emission energy with increasing layer thickness and the corresponding decrease in intensity^44^. The A-exciton energy peak at ~66 nm (1.86 eV) agrees with previous reports of ML-MoS_2_ PL emission^1–3,45^. The weaker B-exciton peak is also well-resolved in the monolayer on SiO_2_ at ~62 nm (~2.00 eV) and arises from the κ-point band splitting due to the valence band spin—orbit coupling. The splitting between the A- and B-excitons in our monolayers is ~14  meV, which is in excellent agreement with the theoretical value (148 meV) for MoS_2_ film^46^. This is strong evidence for the excellent crystallinity of our material.

**Figure 3:**
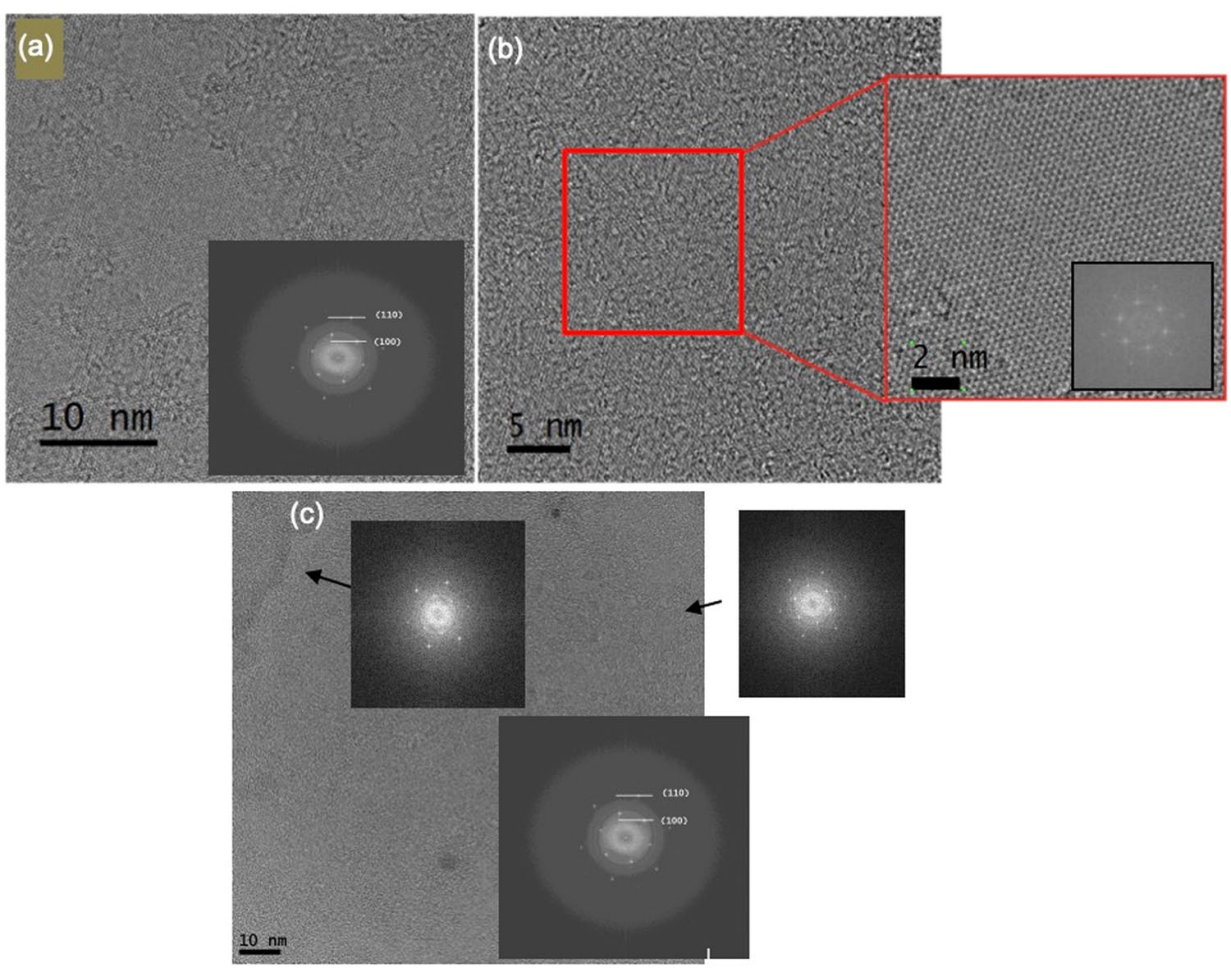
TEM data from different regions of material grown without post-annealing. (**a**) A region exhibiting 2H phase purity, as confirmed by FFT (inset) (**b**) Magnification (red box) and FFT (inset) of crystalline region clearly show the hexagonal lattice pattern, consistent with 2H-MoS_2_. (**c**) TEM image and FFT data (insets) from either side of a grain boundary in a large crystal. FFT of the entire region is also shown (inset, bottom right).

TEM provides further confirmation of crystalline monolayer formation (Fig. 3). FFT patterns confirm the hexagonal crystal structure of the material, complementing the Raman and PL data that show the 2H phase was synthesised.

The TEM images presented in Fig. 3(a,b) demonstrate that a defect-free crystalline structure has been achieved, which is comparable to results obtained from CVD^12,13^. The diffraction patterns shown in FFT (insets) correspond to the (100) and (110) lattice planes of hexagonal MoS_2_^11,12^ with an interplanar d-spacings between neighbouring planes of d_100_ = 0.27 nm and d_110_ = 0.16 nm. These findings are in agreement with the reported spacings for hexagonal MoS_2_^11^ (see Supplementary Fig. S3 for details of calculations).

FFT was performed across a grain boundary of a 100 μm complex-faceted crystal to determine its uniformity, as shown in Fig. 3(c). Previous reports suggest that the butterfly, hourglass and kite morphologies can result from single crystal growth of 2D materials. FFT taken from small regions on either side of the grain boundary were compared to the FFT of the whole 100 × 100 nm region. The three patterns show the same set of six-fold-symmetrical diffraction points and are consistent with single-crystalline monolayer growth^5,13^. These results combined with OM and SEM images suggest that single crystals with lateral sizes up to 100 μm have been synthesised by our method.

## Conclusions

We have demonstrated a simple, single-cycle and single-substrate route to large single-crystal domains (up to 100 μm) of MoS_2_ using a novel Mo precursor that requires no surface pre-treatment. We anticipate that grain size can be further improved by careful control of substrate and sulphurisation parameters. The single-cycle nature of our process and short annealing time could lead to a significant improvement in the cost and volume of large-scale MoS_2_ production.

## Supplementary information

Supporting information

## Data Availability

All data generated or analysed during this study are included in this published article (and its Supplementary Information files).
