# Supplementary material for: A Single-Step Route to Single-Crystal Molybdenum Disulphide (MoS2) Monolayer domains
Source: Sci Rep. 2019 Mar 11;9:4142. doi: 10.1038/s41598-019-40893-z (PMC6411997; doi:10.1038/s41598-019-40893-z)
Supplement: Supplementary file 1 — Supporting information [file 41598_2019_40893_MOESM1_ESM.docx]

A Single-Step Route to Single-Crystal Molybdenum Disulphide (MoS_2_) Monolayer domains

Hamid Khan, Henry Medina, Lee Kheng Tan, Wengweei Tjiu, Stuart A. Boden, Jinghua Teng and Iris Nandhakumar

# Supporting Information

**Table S1.** Deposition parameters of molybdenum-containing precursors

| **Sample no. [substrate]** | **Solvent** | **Conc./g mL^-1^** | **Dip-coat time/min.** | **Temp./** |
| --- | --- | --- | --- | --- |
| T1 [1] | Water | 0.2 | 1 | 40 |
| T2 [1] | Water | 0.2 | 15 | 40 |
| T3 [1] | Water | 0.2 | 60 | 40 |
| T4 [1] | Acetone | 0.2 | 1 | 40 |
| T5 [1] | Acetone | 0.2 | 15 | 40 |
| T6 [1] | Acetone | 0.2 | 60 | 40 |
| T7 [1] | Water | 0.2 | 1 | 25 |
| T8 [1] | Water | 0.2 | 15 | 25 |
| T9 [1] | Water | 0.2 | 60 | 25 |
| T10 [1] | Acetone | 0.2 | 1 | 25 |
| T11 [1] | Acetone | 0.2 | 15 | 25 |
| T12 [1] | Acetone | 0.2 | 60 | 25 |
| T13 [1] | Water | 0.2 | 15 | 50 |
| T14 [1] | Water | 0.2 | 15 | 60 |
| T15 [1] | Water | 0.2 | 15 | 70 |
| T16 [1] | Water | 0.2 | 15 | 80 |
| T17 [1] | Water | 0.2 | 15 | 90 |
| T18 [1] | Water | 0.13 | 15 | 50 |
| T19 [1] | 40% EtOH | 0.2 | 15 | 50 |
| T20 [1] | 40% EtOH | 0.2 | 15 | 50 |
| T18’ [1] | Water | 0.13 | 15 | 70 |
| T19’ [1] | 40% EtOH | 0.13 | 15 | 70 |
| T20’ [1] (crack) | Water | 0.13 | 15 | 70 |
| T21 [1] | Water | 0.13 | 15 | 80 |
| T21’ [1] | Water | 0.13 | 15 | 80 |
| T22 [1] | Water | 0.13 | 15 | 70 |
| T23 (11 nm etch) [1] | Water | 0.13 | 15 | 70 |
| T24 (31 nm etch) [1] | Water | 0.13 | 15 | 70 |
| T25 (45 nm etch) [1] | Water | 0.13 | 15 | 70 |
| T26 [1] | Water | 0.13 | 15 | 80 |
| T27 (=T22) [1] | Water | 0.13 | 15 | 70 |
| T28 [1] | Water | 0.13 | 15 | 80 |
| T28’ [1] | Water | 0.13 | 15 | 80 |
| T29 [2] | Water | 0.13 | 15 | 70 |
| T30 [1] | Water | 0.13 | 15 | 70 |
| T31 [1] | Water | 0.13 | 15 | 80 |
| T32 [2] | Water | 0.13 | 15 | 80 |
| T33 [2] | Water | 0.13 | 15 | 70 |
| T34 [2] | Water | 0.13 | 15 | 70 |
| T35 [1] | Water | 0.13 | 15 | 80 |
| T36 [1] | Water | 0.13 | 15 | 80 |

Precursor deposition parameters. Substrate [1] is SiO_2_/Si, and substrate [2] is c-cut sapphire, which has not been discussed in the main paper. There are additional notes about substrate preparation: T23-25 were prepared with 11, 31 and 45 nm channels created by reactive-ion etching before deposition. T35-36 were prepared by 20-min. pre-treatment of the substrate with 0.2 M aqueous KOH to functionalise the surface. A number of solvents were used for the precursor solution, but water was judged the best one. Increasing dip-coating time from 1 min. to 15 min. resulted in a substantial improvement in coverage, but a further increase to 60 min. resulted in only marginal improvement.

**Table S2.** Sulphurisation and annealing parameters in elemental sulphur

| **Sample** | **Sulphur/mg** | **Gas** | **Gas flow/sccm** | **Temp/** | **Time/min** | **Post-anneal temp/** | **Time/min.** |
| --- | --- | --- | --- | --- | --- | --- | --- |
| T1 | 1600 | N_2_ | 100 | 800 | 10 |  |  |
| T2 | 1600 | N_2_ | 100 | 800 | 10 |  |  |
| T3 | 1600 | N_2_ | 100 | 800 | 10 |  |  |
| T4 | 1600 | N_2_ | 100 | 800 | 10 |  |  |
| T5 | 1600 | N_2_ | 100 | 800 | 10 |  |  |
| T6 | 1600 | N_2_ | 100 | 800 | 10 |  |  |
| T7 | 1600 | N_2_ | 100 | 800 | 10 |  |  |
| T8 | 1600 | N_2_ | 100 | 800 | 10 |  |  |
| T9 | 1600 | N_2_ | 100 | 800 | 10 |  |  |
| T10 | 1600 | N_2_ | 100 | 800 | 10 |  |  |
| T11 | 1600 | N_2_ | 100 | 800 | 10 |  |  |
| T12 | 1600 | N_2_ | 100 | 800 | 10 |  |  |
| T13 | 1600 | N_2_ | 100 | 800 | 10 |  |  |
| T14 | 1600 | N_2_ | 100 | 800 | 10 |  |  |
| T15 | 1600 | N_2_ | 100 | 800 | 10 |  |  |
| T16 | 1600 | N_2_ | 100 | 800 | 10 |  |  |
| T17 | 1600 | N_2_ | 100 | 800 | 10 |  |  |
| T18 | 1600 | N_2_ | 100 | 800 | 10 |  |  |
| T19 | 1600 | N_2_ | 100 | 800 | 10 |  |  |
| T20 | 1600 | N_2_ | 100 | 800 | 10 |  |  |
| T18’ | 1600 | N_2_ | 100 | 800 | 10 |  |  |
| T19’ | 1600 | N_2_ | 100 | 800 | 10 |  |  |
| T20’ | 1600 | N_2_ | 100 | 800 | 10 |  |  |
| T21 | 1200 | 5% H_2_ | 60 | 800 | 10 |  |  |
| T21’ | 200 | 5% H_2_ | 60 | 800 | 10 |  |  |
| T22 | 2000 | N_2_ | 100 | 800 | 10 | 1000 | 60 |
| T23 | 2000 | N_2_ | 100 | 800 | 10 |  |  |
| T24 | 2000 | N_2_ | 100 | 800 | 10 |  |  |
| T25 | 2000 | N_2_ | 100 | 800 | 10 |  |  |
| T26 | 2000 | N_2_ | 100 | 800 | 10 |  |  |
| T27 | 2000 | N_2_ | 100 | 800 | 10 | 1000 | 60 |
| T28 | 1200 | 5% H_2_ | 60 | 800 | 10 |  |  |
| T28’ | 200 | 5% H_2_ | 60 | 600 | 10 |  |  |
| T29 | 2000 | Ar | 100 | 800 | 10 |  |  |
| T30 | 2000 | Ar | 100 | 800 | 10 |  |  |
| T33 | 2000 | Ar | 100 | 800 | 10 | 1000 | 60 |
| T34 | 2000 | Ar | 100 | 800 | 10 | 1000 | 60 |
| T35 |  | | | | | 1000 | 60 |
| T36 |  |  |  |  |  | 1000 | 60 |

Sulphurisation in elemental sulphur. In most cases, 1600-2000 mg sulphur was used. In later experiments in a smaller tube furnace, these amounts were inappropriate, and so the amount was reduced first to 1200 mg and then 200 mg to mitigate hazards related to blockage of the exhaust. Where 5%H_2_ is shown, the sample was annealed in a 90:10 mixture of argon (54 sccm) and hydrogen (6 sccm) up to 500℃, and then in argon thereafter. Initial annealing temperatures were 600-800 ℃. Post-annealing parameters are shown where it was performed, but these results are not discussed in detail in the paper for reasons to do with quality.

Thermal decomposition of ammonium heptamolybdate tetrahydrate was studied by thermogravimetric analysis (TGA) under furnace conditions. A small amount (~5 mg) of precursor was heated from room temperature to 750 ℃ at 50 ℃/min. in nitrogen (100 sccm/0.1 slm). The vendor specified 81.0-83.3% base MoO_3_ in the as-purchased precursor.


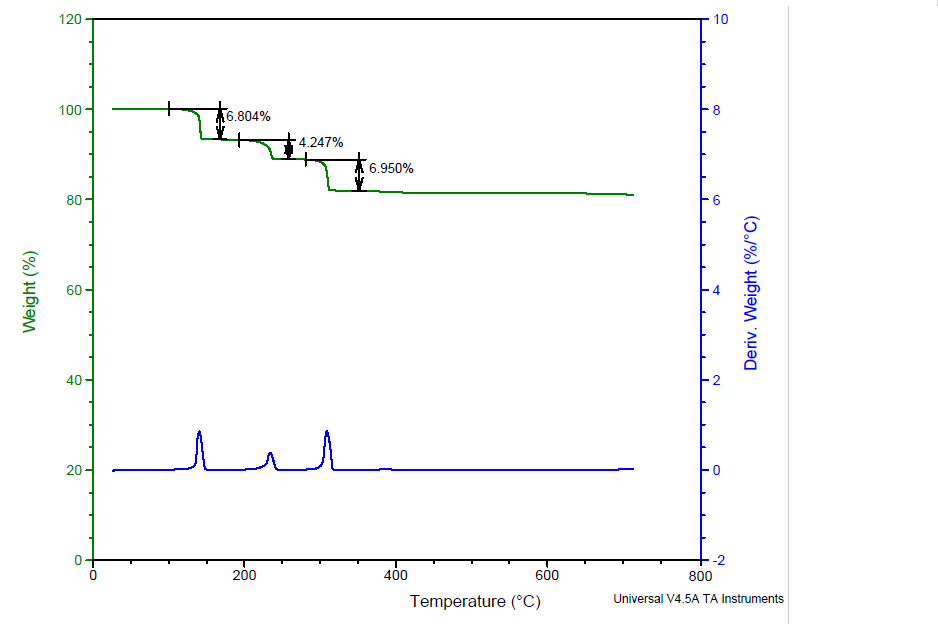


**Figure S1:** The TGA trace shows a stepped decline in the mass of precursor starting from around 140℃ to 310 ℃. The trace of derivative weight percent with respect to temperature resolves the exact transition temperatures.

The thermal decomposition of the precursor under furnace conditions was used to understand the reaction chemistry during sulphurisation and annealing. The end mass was 81.9% of the initial, consistent with the vendor’s specified percentage of base MoO_3_ in the as-bought precursor. Decomposition proceeds via three steps. These steps have been reported previously as loss of ammonia and water vapour and are shown below.^1^

**Scheme S3.** Decomposition of ammonium heptamolybdate tetrahydrate

1. 140℃: loss of 6.80% mass


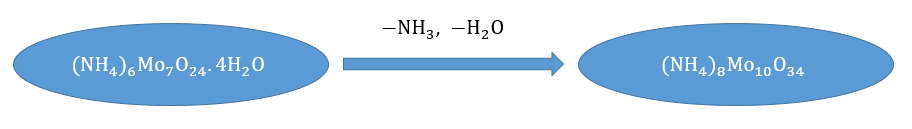


1. 240 ℃: loss of 4.25% mass


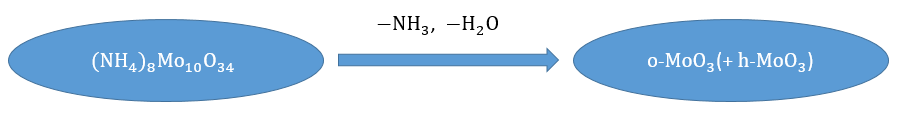


1. 310 ℃: loss of 6.95% mass


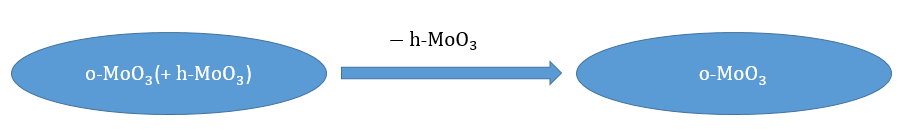


In i), the mass loss to 93.2% at 140 ℃ corresponds exactly with reports from the literature. In ii), the mass loss to 88.9% at 230 ℃ corresponds very closely to the literature reports of 88.7% as a mixture of hexagonal and orthorhombic MoO_3. In iii), the mass loss to 81.9% at 310 ℃ again corresponds very closely to the literature reports of 81.2% as the metastable hexagonal phase transforms into the thermodynamic orthorhombic phase. The overall decomposition is not accompanied by reduction of molybdenum. The metal centres in ammonium heptamolybdate are in the +6 OS, and so is the metal centre in MoO_3_.

The product of thermal decomposition is orthorhombic , a phase that is stable up to ~800. This is instructive for the sulphurisation step. Sulphur evaporates at 445 , so under the temperature of the central heating zone of the reaction tube, sulphur vapour passes over the -phase, and the overall reaction proceeds as in Equation 1:^2^

2 MoO_3_ + 7 S 🡪 2 MoS_2_ + 3 SO_2_ (Eq. 1)

Figure S2: Raman intensity maps of 120x100 μm regions of post-annealed samples: (left) E_2g_ intensity map and (right) A_1g_ intensity map; scale bar = 20 μm.

**
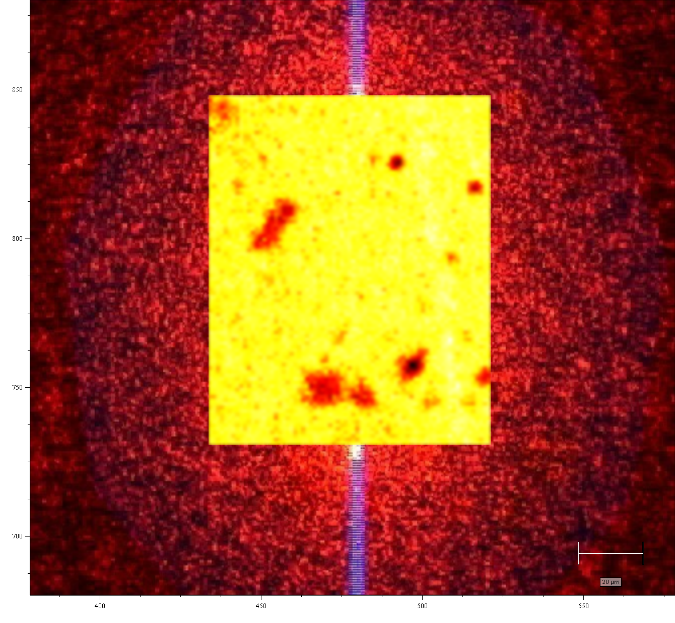

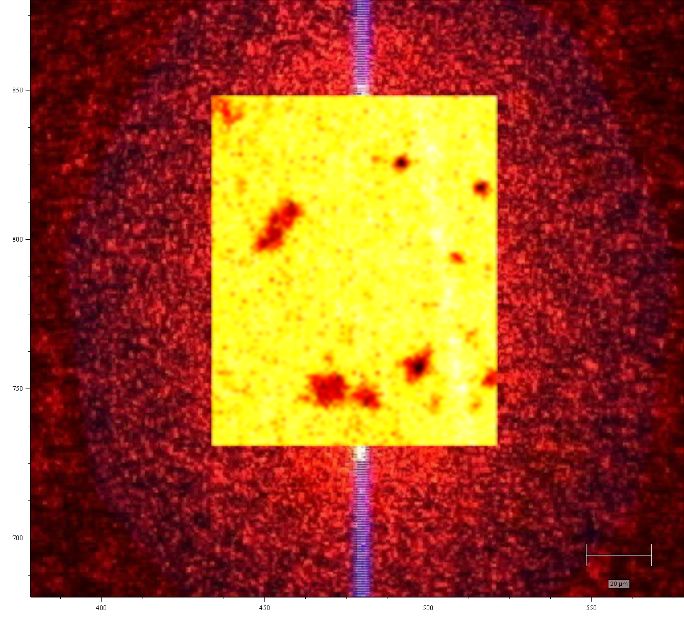
**

Figure S3: The characteristic d-spacing of the (100) lattice plane can be calculated by appropriate analytical software. We used GMS Digital Micrograph. The procedure is outlined below:

(b)

(a)


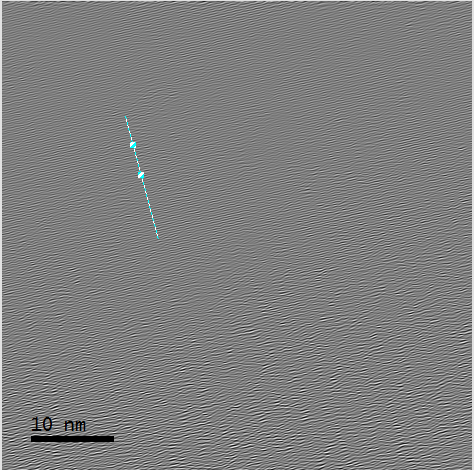


(d)

(c)


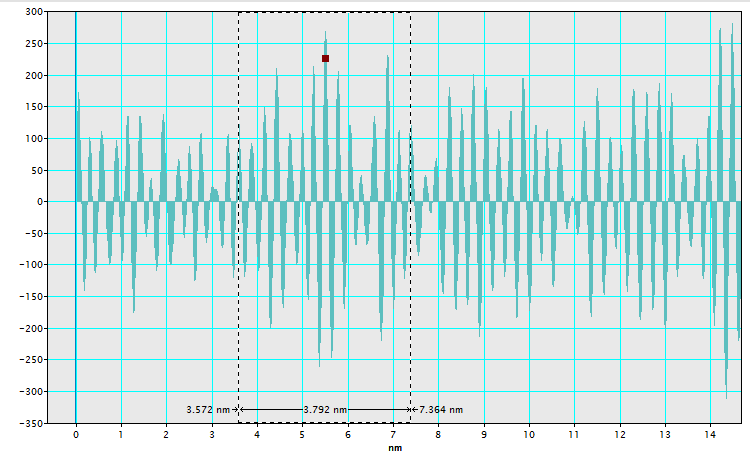


- A region of interest is selected (a) and an FFT taken (b).
- Two diametrically-opposite spots in the same diffraction pattern are masked and the unmasked areas removed from the image (c).
- An inverse FFT of the masked area is taken to yield an image of the lattice planes in the crystal (d). A line profile is taken perpendicular to the lattice planes.
- The line profile is plotted (e). To determine the d-spacing, two points are selected that are of roughly equal height on the +y-axis. The distance in +x between these two points is read as 3.792 nm. The number of “spaces” between the two points is counted (14). Dividing the distance by the number of spaces gives the d-spacing between two adjacent lattice planes.

(3.792 nm)/14=0.271 nm

## References

1. Kovacs, T. N.; Hunyadi, D.; de Lucena, A. L. A.; Szilagyi, I. K. *J. Therm. Anal. Calorim.* **2016,** 124, (2), 1013-1021.

2. Li, X. L.; Li, Y. D. *Chem. Eur. J* **2003,** 9, (12), 2726-2731.
